# The Relationship between Cyber Violence Victimization and Adverse Childhood Experiences

**DOI:** 10.1192/j.eurpsy.2024.1215

**Published:** 2024-08-27

**Authors:** S. E. Ilgin, Ö. Yanartaş, O. Polat

**Affiliations:** ^1^Psychiatry, Marmara University Research & Training Hospital; ^2^Forensic Medicine, Acibadem University, Istanbul, Türkiye

## Abstract

**Introduction:**

Nowadays, following the increasing digital technologies, the cyber aspect of violence is becoming more common (Willard, Journal of Adolescent Health 2007; 41) At this point, we can think that cyber violence victimization, environmental factors such as childhood traumas, exposure to violence, and moreover psychological and biological factors play a role (Fan et al., Front. Psychol 2921;12)

**Objectives:**

Our aim was to conduct a study to determine the prevalence of cyber violence victimization among university students and to examine whether this prevalence would be related to negative childhood traumas.

**Methods:**

We reached university students in many cities of Turkey through an online survey between 01 January 2023 and 31 March 2023. A total of 600 students participated. In our survey, we used the Turkish forms of the Childhood Adverse Experiences Scale and the Cyber Victimization Scale, for which sociodemographic data, validity and reliability studies have been completed in Turkish.

**Results:**

University students from 8 different provinces participated in our study. According to the results of our study, it was evaluated that university students who had negative childhood experiences were more likely to become cyber victims. We are exposed to many traumas from the moment we are born, and this exposure is the relationship with the mother, then relatives, friends, teachers, colleagues, etc. It continues throughout life through relationships. There is a moderate relationship between victimization of blocking and damaging behaviors in cyberspace and negative childhood relationships (*r* = 0.304), a low level relationship between victimization of sexual bullying in cyberspace and negative childhood relationships (*r* = 0.289), and victimization of spreading rumors in cyberspace. A low degree of correlation (*r* = 0.277) was found between and negative childhood relationships. (*p* <0.05)

**Conclusions:**

The widespread use of technology today facilitates the digitalization of violence, as in every field. As a result, cyber violence, like other types of violence, may be associated with childhood traumas, and this can be prevented by being exposed to the least trauma during childhood, and moreover, if we look from Bronfenbrenner’s perspective, if the next generation grows up in a suitable ecological environment, cyber violence victimization can be prevented to that extent.

**Disclosure of Interest:**

None Declared

